# Quality Evaluation of Ophiopogonis Radix from Two Different Producing Areas

**DOI:** 10.3390/molecules24183220

**Published:** 2019-09-04

**Authors:** Mengxia Tan, Jiali Chen, Chengcheng Wang, Lisi Zou, Shuyu Chen, Jingjing Shi, Yuqi Mei, Lifang Wei, Xunhong Liu

**Affiliations:** College of Pharmacy, Nanjing University of Chinese Medicine, Nanjing 210023, China (M.T.) (J.C.) (C.W.) (L.Z.) (S.C.) (J.S.) (Y.M.) (L.W.)

**Keywords:** Ophiopogonis Radix, different producing areas, multiple bioactive constituents, multivariate statistical analysis

## Abstract

Ophiopogonis Radix, also known as Mai-dong in Chinese, was a commonly used traditional Chinese medicine (TCM) and functional health food. Two products of Ophiopogonis Radix are largely produced in the Sichuan and Zhejiang province, which are called “Chuan maidong (CMD)” and “Zhe maidong (ZMD)” respectively. To distinguish and evaluate the quality of CMD and ZMD, an analytical method based on ultra-fast performance liquid chromatography coupled with triple quadrupole-linear ion trap mass spectrometry (UFLC-QTRAP-MS/MS) was established for simultaneous determination of 32 constituents including 4 steroidal saponins, 3 homisoflavonoids, 15 amino acids, and 10 nucleosides in 27 Mai-dong samples from Sichuan and Zhejiang. Furthermore, principal components analysis (PCA), partial least squares discriminant analysis (PLS-DA), *t*-test, and grey relational analysis (GRA) were applied to discriminate and evaluate the samples from Sichuan and Zhejiang based on the contents of 32 constituents. The results demonstrated that the bioactive constituents in CMD and ZMD were significantly different, and CMD performed better in the quality assessment than ZMD. This study not only provides a basic information for differentiating CMD and ZMD, but offers a new insight into comprehensive evaluation and quality control of Ophiopogonis Radix from two different producing areas.

## 1. Introduction

Ophiopogonis Radix, the root of *Ophiopogon japonicas* Ker-Gawl. (Liliaceae), was applied as a traditional Chinese medicine (TCM) documented in the Chinese Pharmacopoeia [1]. It has been used to nourish the *yin*, promote body fluid production, moisten the lung, and clear away heartburn (according to the traditional Chinese medicine theory) [2]. Modern phytochemical research has revealed that Ophiopogonis Radix contains various components including steroidal saponins, homoisoflavonoids, amino acids, nucleosides, and polysaccharides [2,3,4,5]. Pharmacological studies have demonstrated that steroidal saponins and homoisoflavonoids possess anti-oxidation, anti-inflammation, immunomodulation, cardiovascular protection, and anticancer and antitussive effects [6,7,8,9,10]. In addition, studies suggested that amino acids and nucleosides have wide physiological activities, such as enhancing immunity, regulating the central nervous system, and improving cell metabolism [11,12].

In China, Ophiopogonis Radix was largely produced in the Sichuan and Zhejiang province, which could explain why they were named as Chuan maidong (CMD) and Zhe maidong (ZMD) respectively. Although both of them originated from *Ophiopogon japonicas*, their appearance and plant growth cycles are different. CMD is demanded one year to harvest, while ZMD is harvested after two or three years. The phenomenon may result in the differences of the accumulation of chemical constituents in CMD and ZMD. Due to the long growth cycle and the high price, the market of ZMD has shrunk in recent years. CMD becomes the primary commodity of Ophiopogonis Radix [13]. Besides this, the differences of the chemical constituents and pharmacological activities in CMD and ZMD have been discovered by a few studies [13,14,15]. However, the research on distinction and comprehensive evaluation of Ophiopogonis Radix grown in two different areas remains still limited.

At present, various analytical methods have been reported for the quality evaluation and control of Ophiopogonis Radix, such as high-performance liquid chromatography combined with ultraviolet detector or evaporative light scattering detector (HPLC-UV/ELSD) and liquid chromatography-mass spectrometer (LC-MS) [14,15,16,17,18,19]. However, more attention was paid to quantitative analysis of several steroidal saponins or homoisoflavonoids because of poor UV absorption or low content [16,18,19]. Few studies reported that steroidal saponins, homoisoflavonoids, amino acids, and nucleosides were simultaneously determined based on LC-MS [19]. Hence, it is crucial to establish a relatively reliable analytical method to differentiate CMD and ZMD, and comprehensively evaluate the quality of them combined with multiple bioactive constituents.

The aim of our study was to distinguish and evaluate the quality of CMD and ZMD based on simultaneous determination of multiple bioactive constituents combined with multivariate statistical analysis. A reliable method based on ultra-fast performance liquid chromatography coupled with triple quadrupole-linear ion trap mass spectrometry (UFLC-QTRAP-MS/MS) was developed to simultaneously determine the contents of 32 components in Ophiopogonis Radix from Sichuan and Zhejiang. Furthermore, principal component analysis (PCA), partial least squares discriminant analysis (PLS-DA), and *t*-test were applied to discriminate and reveal differential constituents between CMD and ZMD [20,21,22,23]. Grey relational analysis (GRA) was employed for the quality evaluation of Ophiopogonis Radix according to the correlation relationship between the contents of components detected and the samples [20,21,22,23]. The established method might lay the foundation for comprehensive evaluation and quality control of Ophiopogonis Radix from two different producing areas, as well as provide a basic data to discriminate CMD and ZMD.

## 2. Results

### 2.1. Optimization of Extraction Conditions

The extraction solvent, time, and solid-liquid ratio were important to the extraction of target chemicals in Ophiopogonis Radix. To obtain the appropriate extraction efficiency of Ophiopogonin D, Methylophiopogonanone A, Arginine, and Uridine, the extraction solvent (50% methanol/ethanol, 70% methanol/ethanol, methanol/ethanol, *v*/*v*), the extraction time (30, 60, and 90 min), and the extraction solid-liquid ratio (1:10, 1:20 and 1:30, *v*/*v*) were performed for the single factor test. Finally, the optimal extraction method of UFLC-QTRAP-MS/MS was 1.0 g sample powders were extracted by 70% methanol (*v*/*v*, 30 mL) with ultrasonic machine for 60 min.

### 2.2. Optimization of Ultra-fast Performance Liquid Chromatography (UFLC) Conditions

The UFLC chromatographic conditions, such as column, mobile phase, and column temperature were optimized to achieve the higher separation efficiency and the better peak shape of target compounds in Ophiopogonis Radix. Therefore, three chromatographic columns of Thermo Acclaim™ RSLC 120 C_18_ column (2.1 mm × 150 mm, 2.2 μm) (Thermo Scientific, Waltham, MA, USA), XBridge^®^ C_18_ column (4.6 mm × 100 mm, 3.5 μm) (Waters, Wexford, Ireland), and Synergi™ Hydro-RP100Å column (2.0 mm × 100 mm, 2.5 μm) (Phenomenex, California, USA) were investigated in the experiment. The results showed that Synergi™ Hydro-RP100Å column (2.0 mm × 100 mm, 2.5 μm) could improve the separation degree and sensitivity of the detected components. Besides, four mobile phase systems (water-methanol, water-acetonitrile, 0.1% formic acid water solution-0.1% formic acid methanol solution, and 0.1% formic acid water solution-0.1% formic acid acetonitrile solution), flow rates (0.3, 0.35 and 0.4 mL/min), and column temperatures (25, 30, 35 °C) were examined and compared. Ultimately, 0.1% formic acid solution-0.1% formic acid acetonitrile with 0.4 mL/min at 30 °C on Synergi™ Hydro-RP100Å column (2.0 mm × 100 mm, 2.5 μm) was optimized and applied.

### 2.3. Optimization of Mass Spectrometry (MS) Conditions

**Figure 1:**
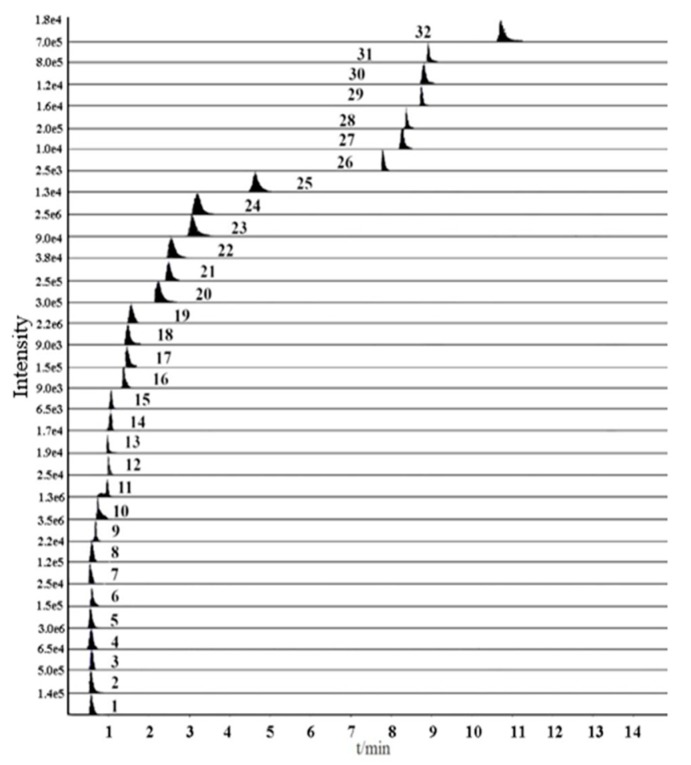
Representative extract ions chromatograms (XIC) of multi-reaction monitoring (MRM) chromatograms of 32 investigated compounds. (Compounds were shown in Table 1).

To develop a sensitive and accurate quantitative method, individual solutions of all standards were detected by a full-scan mass spectrometry method in both positive and negative modes. After trial and error inspection, steroidal saponins, amino acids, and nucleosides have good sensitivity and intensity in the positive ion mode. However, the responses of homoisoflavonoids were significantly higher in the negative ion mode than in the positive ion mode. Thus, the ESI^+^ and ESI^−^ mode were simultaneously adopted in this study. Although the retention times of some compounds were similar, they could be accurately quantified based on different precursor and product ion pairs. The optimum details including retention time (t_R_), precursor and product ions, declustering potential (DP) and collision energy (CE) of 32 compounds were listed in Table 1. Multi-reaction monitoring (MRM) of 32 compounds were showed in Figure 1.

### 2.4. Method Validation

All method validations of quantification were performed by the established UFLC-QTRAP-MS/MS method. The detailed results of each method validation were presented in Table S1. Each standard calibration curve was constructed by plotting the peak areas (*Y*) against the corresponding concentrations (*X*). All analytes showed good linearity with appropriate determination coefficients (*r* > 0.9991). The ranges of LODs and LOQs were 0.11–2.92 ng/mL and 0.37–9.73 ng/mL, respectively. The RSDs of intra-day and inter-day variations ranged from 0.45% to 4.64% and 1.58% to 4.80%, respectively. The RSDs of repeatability and stability test were less than 4.56% and 4.57%, respectively. The overall recoveries varied from 95.58% to 102.46%, with RSDs < 4.45%. The slope ratio values of the matrix curve to pure solution curve were between 0.93 and 1.08, indicating that the matrix effect on the ionization of analytes was not obvious under optimized conditions.

### 2.5. Quantitative Analysis of Samples

**Figure 2:**
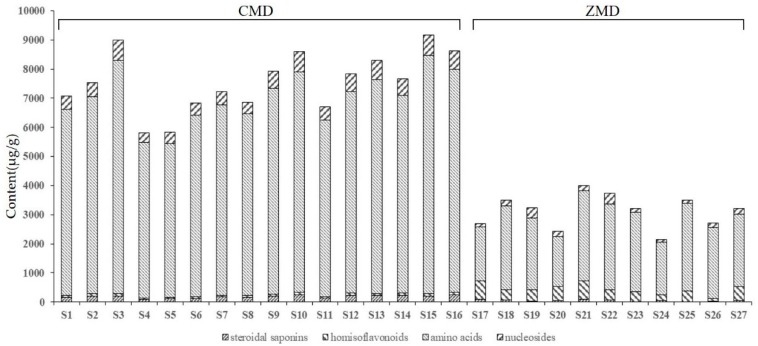
The contents of four kinds of chemical components in Ophiopogonis Radix from different producing areas.

The validated analytical method of UFLC-QTRAP-MS/MS was successfully applied to simultaneously determine 32 components (4 steroidal saponins, 3 homisoflavonoids, 15 amino acids, and 10 nucleosides) in Ophiopogonis Radix. The quantitative results of 32 components were presented in Table S2. All Ophiopogonis Radix samples were rich in amino acids, the content of total amino acids varied from 1714.90 to 8190.81 μg/g, which accounted for more than 65% of the total contents of all analytes tested in this study. In addition, the contents of Arginine (**4**), Threonine (**6**), Serine (**7**), Alanine (**8**), and Proline (**9**) were the relatively higher in the detected amino acids. As for nucleosides, the total content of them ranged from 77.84 to 710.20 μg/g, among which, Uridine (**17**) and Guanosine (**22**) contents occupied more than 75% in these tested nucleosides. In terms of secondary metabolites, the ranges of 4 steroidal saponins were 0.30–178.20 μg/g in CMD and 0.00–85.50 μg/g in ZMD respectively. 3 homisoflavonoids contents ranged from 0.79 to 60.90 μg/g and 0.41 to 393.00 μg/g. Besides, Ophiopogonin D (**27**) was not detected in samples from Zhejiang province. The contents of Methylophiopogonanone A (**30**) and Methylophiopogonanone B (**31**) in CMD were below ZMD, the ratio of **30** and **31** in CMD was opposite to ZMD. Combined with the results of metabolites detected, Figure 2 suggested that the content of amino acids, nucleosides and steroidal saponins in CMD was obviously higher than ZMD. However, homisoflavonoids content in CMD was less than ZMD. The results clearly demonstrated that the total contents of 32 chemical components in CMD samples were significantly different from ZMD.

### 2.6. PCA of Samples

**Figure 3:**
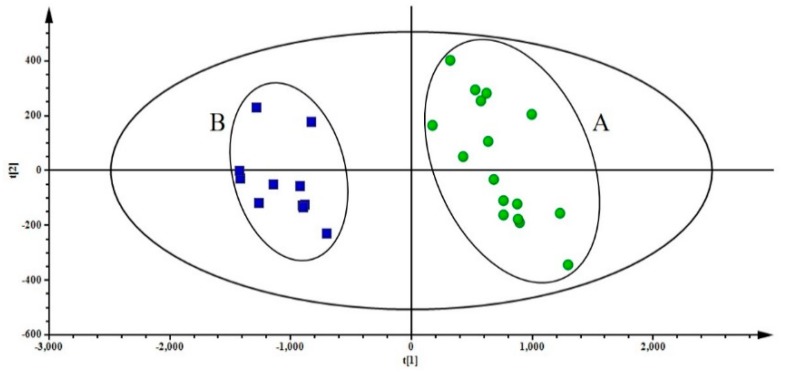
The PCA chromatogram of Chuan maidong (CMD) (**A**) and Zhe maidong (ZMD) (**B**).

According to the contents of 32 components, PCA was performed to classify and distinguish Ophiopogonis Radix from Sichuan and Zhejiang (PR China). The first two principal components accounted for more than 90%, which could be used to represent overall information of samples (R2X[1] = 0.908, R2X[2] = 0.0377), and be applied to discriminate CMD samples (group A) and ZMD samples (group B). The PCA scores plot showed that CMD and ZMD samples were divided into two clusters, samples of group A were mainly gathered in the positive axis of t[1], while samples of group B were located in the negative axis of t[1] (Figure 3). The phenomenon demonstrated that there are significant differences in constituents between CMD and ZMD.

### 2.7. Partial Least Squares Discriminant Analysis (PLS-DA) of Samples

**Figure 4:**
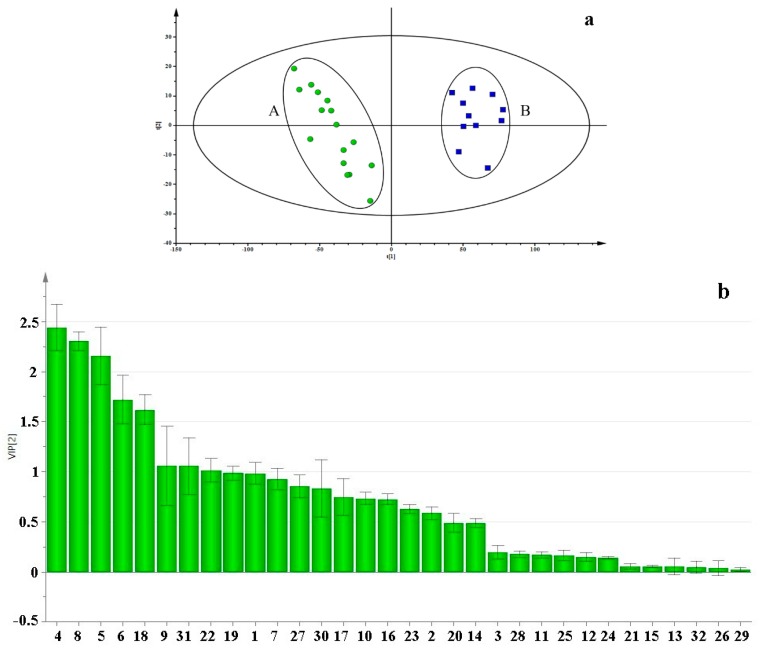
The partial least squares discriminant analysis (PLS-DA) score scatter plot (**a**) and variable importance in the projection (VIP) (**b**) of Chuan maidong (CMD) (**A**) and Zhe maidong (ZMD) (**B**).

PLS-DA, a supervised pattern recognition method, was employed to differentiate CMD and ZMD, and to find the differential constituents with variable importance in the projection (VIP) values. The PLS-DA score scatter plot and VIP values are shown in Figure 4a,b. The established PLS-DA model showed good fitness (R2X = 0.914, R2Y = 0.959) and predictability (Q2 = 0.945). CMD and ZMD samples were separated into two groups along PC1 axis. The result illustrated that the differences of constituents between CMD and ZMD are remarkable. The VIP values were used to describe the contribution of each variable to the model and explore the differential constituents for the classification of CMD and ZMD. The VIP-value cutoff usually was set at 1.0, eight differential constituents were picked out to discriminate CMD and ZMD, including Arginine (**4**), Threonine (**5**), Aspartic acid (**6**), Alanine (**8**), Proline (**9**), Leucine (**18**), Guanosine (**22**), and Methylophiopogonanone B (**31**). These constituents could be selected as potential chemical markers to distinguish Ophiopogonis Radix from two different producing areas.

### 2.8. t-Test of Samples

**Figure 5:**
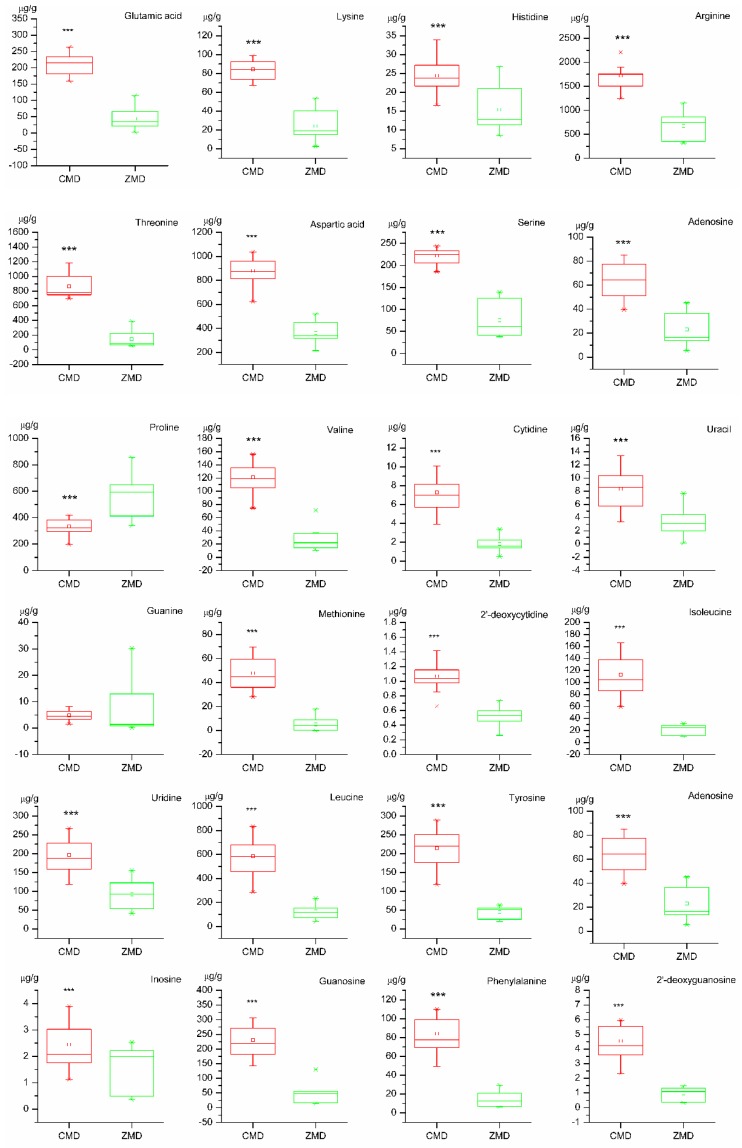

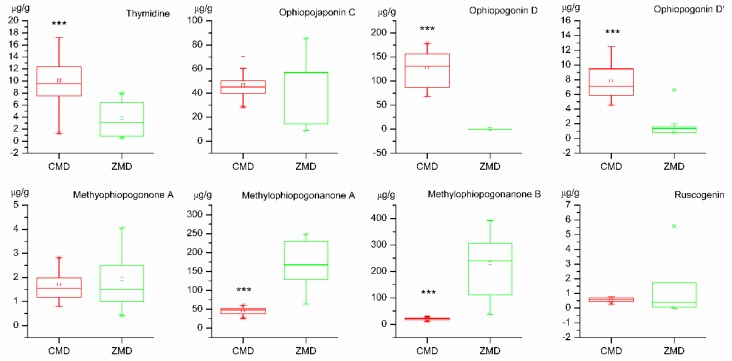
The box plot of 32 compounds contents in Chuan maidong (CMD) and Zhe maidong (ZMD) (*** *p* < 0.001).

The contents of detected bioactive constituents were analyzed by *t*-test to evaluate the variation of 32 constituents in CMD and ZMD, the values of *p* less than 0.05 were considered remarkably different. 28 bioactive constituents, were analyzed in this study, were significantly different in Ophiopogonis Radix from two different areas. As shown in Figure 5, more than two-thirds of the bioactive constituents in CMD were higher than ZMD (*p* < 0.001). However, Methylophiopogonanone A (**30**) and Methylophiopogonanone B (**31**) displayed higher level in ZMD than CMD (*p* < 0.001), and the content of Methylophiopogonanone A was higher than Methylophiopogonanone B in CMD, while the contents of these components in ZMD was opposite to CMD. There were no significant differences of Guanine (**13**), Ophiopojaponin C (**26**), Methylophiopogonone A (**29**), and Ruscogenin (**32**) between CMD and ZMD samples.

### 2.9. Grey Relational Analysis (GRA) of Samples

GRA is an impact measurement method in Grey system theory that analyzes uncertain relations between one main factor and all other factors in a given system. Hence, GRA was carried out to comprehensively evaluate CMD and ZMD based on the contents of 32 bioactive constituents. The GRA results including the grey comprehensive evaluation values (the relative correlation degree, *r_i_*) and quality-ranking were displayed in Table 2. The relative correlation degree represented the relative correlation relationship between the contents of components and the samples. The sample with the relatively higher relative correlation degree possessed a better quality. According to *r_i_* values of samples, the quality of CMD was better than that of ZMD.

## 3. Discussion

An efficient and reliable method based on UFLC-QTRAP-MS/MS was developed to simultaneously determinate multiple bioactive constituents including 4 steroidal saponins, 3 homisoflavonoids, 15 amino acids, and 10 nucleosides in Ophiopogonis Radix from Sichuan and Zhejiang provinces. The PCA result showed that the samples of CMD and ZMD were obviously divided into two clusters (Figure 3 and Figure 4a). The PLS-DA, VIP values, and t-test revealed that the bioactive constituents in CMD and ZMD were significantly different, such as Arginine (**4**), Threonine (**5**), Aspartic acid (**6**), Alanine (**8**), Proline (**9**), Leucine (**18**), Guanosine (**22**), and Methylophiopogonanone B (**31**), which may be considered as the chemical marker to discriminate and control the quality of CMD and ZMD (Figure 4b and Figure 5). Besides this, the contents of compounds **4**, **5**, **6**, **8**, **18,** and **22** in CMD were remarkably higher than ZMD, while **9** and **31** were lower. These differential constituents could be used to explain that the immunomodulatory and antioxidant activity are different in Ophiopogonis Radix from different producing areas [8,9,13]. Moreover, the quality of CMD was better than ZMD based on *r_i_* values of samples (Table 2), demonstrated that the growth period and the geographical area did influence the accumulation of bioactive constituents. Overall, the UFLC-QTRAP-MS/MS method combined with multivariate statistical analysis could provide the basic information to discrimination and quality evaluation of Ophiopogonis Radix from two different producing areas.

## 4. Materials and Methods

### 4.1. Plant Materials

S1~S16 were CMD, collected from Sichuan Province, PR China. S17~S27 were ZMD, collected from Zhejiang Province, PR China. All the samples were authenticated by Professor Xunhong Liu (Nanjing University of Chinese Medicine, Nanjing, PR China), and were deposited in the laboratory of Chinese medicine identification, Nanjing University of Chinese Medicine. Detailed information was shown in Table 3.

### 4.2. Chemicals and Reagents

The standards of Glutamic acid (**1**), Lysine (**2**), Histidine (**3**), Arginine (**4**), Threonine (**5**), Aspartic acid (**6**), Serine (**7**), Alanine (**8**), Proline (**9**), Valine (**10**), Cytidine (**11**), Uracil (**12**), Guanine (**13**), 2′-deoxycytidine (**15**), Isoleucine (**16**), Uridine (**17**), Leucine (**18**), Tyrosine (**19**), Adenosine (**20**), Inosine (**21**), Guanosine (**22**), Phenylalanine (**23**), 2′-deoxyguanosine (**24**), and Thymidine (**25**) were purchased from Shanghai Yuanye Bio-Technology Co., Ltd. (Shanghai, China). Ophiopojaponin C (**26**), Ophiopogonin D (**27**), Ophiopogonin D′ (**28**), Methylophiopogonone A (**29**), Methylophiopogonanone A (**30**), and Methylophiopogonanone B (**31**) were purchased from Nanjing Liangwei biotechnology Co., Ltd. (Nanjing, China). Methionine (**14**) was obtained from Chengdu Chroma Biotechnology Co., Ltd. (Chengdu, China). Ruscogenin (**32**) was offered by Shanghai nature standard Bio-Technology Co., Ltd. (Shanghai, China). The purities of all compounds were greater than 98%, checked by HPLC analysis. The structures of the 32 standards were shown in Figure S1. Formic acid, acetonitrile and methanol of HPLC grade (Merck, Darmstadt, Germany). Ultrapure water was prepared using a Milli-Q water purification system (Millipore, Bedford, MA, USA). All the other chemicals and solvents were of analytical grade.

### 4.3. Preparation of Standard Solutions

A mixed standard stock solution containing 32 reference standards was prepared with 70% methanol and their concentrations were as following: 1.086 (**1**), 0.986 (**2**), 1.022 (**3**), 0.926 (**4**), 0.812 (**5**), 1.087 (**6**), 0.992 (**7**), 1.044 (**8**), 0.587 (**9**), 0.958 (**10**), 1.017 (**11**), 1.035 (**12**), 0.986 (**13**), 0.525 (**14**), 1.084 (**15**), 0.650 (**16**), 1.004 (**17**), 0.832 (**18**), 1.004 (**19**), 0.568 (**20**), 0.877 (**21**), 0.757 (**22**), 1.061 (**23**), 0.749 (**24**), 1.103 (**25**), 1.015 (**26**), 1.115 (**27**), 0.825 (**28**), 1.250 (**29**), 0.985 (**30**), 1.050 (**31**), 0.890 (**32**) mg/mL, then diluted with 70% methanol to different concentrations to generate the calibration curves. All of the solutions were stored at 4 °C before LC-MS analysis.

### 4.4. Preparation of Sample Solutions

Sample powders (1.0 g) were accurately weighed and ultrasonically extracted with 30 mL 70% methanol for 60 min. After cooling down at room temperature, 70% methanol was added for the lost weight. The extraction was subsequently centrifuged at 12,000 r/min for 10 min and the supernatant was stored at 4 °C and filtered through a 0.22 μm membrane (Jinteng laboratory equipment Co., Ltd., Tianjin, China) prior to injection of LC-MS analysis.

### 4.5. Chromatographic and Mass Spectrometric Conditions

The chromatographic analysis of Ophiopogonis Radix was performed on an SIL-20A XR system (Shimadzu, Kyoto, Japan). The separation was conducted by a Synergi™ Hydro-RP100Å column (2.0 mm × 100 mm, 2.5 μm) at 30 °C and injection volume was 2 μL. The mobile phase contained 0.1% aqueous formic acid (A) and 0.1% aqueous acetonitrile (B) at 0.4 mL/min flow rate with the following gradient elution: 0–5 min, 98% A; 5–6 min, 98–40% A; 6–8 min, 40–20% A; 8–10 min, 20% A; 10–12 min, 20–98% A; 12–15 min, 98% A.

The mass spectrometry detection was performed using an API4500 triple quadrupole mass (AB SCIEX, Framingham, MA, USA) equipped with an electrospray ionization (ESI) source operating under both positive and negative ion modes. The parameters of MS were set as follows: ionization temperature (TEM): 650 °C, spray voltage: 5500 V (positive) and −4500 V (negative); GSI flow rate: 65 L/min; CUR flow rate: 30 L/min.

### 4.6. Validation of the Method

The calibration curves of 32 compounds were achieved by plotting the peak areas (*Y*) against the corresponding concentrations (*X*). The regression equation, correlation coefficient, and linear range were calculated; the limit of quantification (LOQ) and limit of detection (LOD) of each analyte were measured at signal-to-noise ratio (S/N) of about 10 and 3, respectively. Intra- and inter-day precision were determined in the standard solution six times within a single day and 3 consecutive days. The relative standard deviation (RSD) of the peak area was used to measure precision of the developed method. To evaluate the repeatability, S1 was parallelly divided into six parts and extracted by 70% methanol. Then they were analyzed by LC-MS. The prepared S1 solution was analyzed at 0, 2, 4, 8, 12, and 24 h for testing the stability. A recovery test was used to evaluate the established method by the standards addition method. Three levels of 32 standards (approximately equivalent to 80%, 100%, and 120% of each analyte) were added into S1. The mixture was extracted by methanol and analyzed as the method mentioned above. The recovery percentages were calculated by the following equation: (total detected amount − original amount) / added amount × 100%. The matrix effect was evaluated by the slope ratio of matrix-matched solutions and calibration curves of standards (slope matrix / slope solvent) [12,24].

### 4.7. Multivarite Statistical Analysis

PCA, an unsupervised pattern recognition method, has been used for quality control of traditional Chinese medicine. According to the contents of 32 components, PCA was applied to classify and distinguish Ophiopogonis Radix from different habitats by Simca-P 13.0 software (Umetrics AB, Umea, Sweden). In addition, PLS-DA was performed to differentiate CMD and ZMD and find the differential constituents with variable importance in the projection (VIP) values. Besides, all detected components data were statistically analyzed by *t*-test (SPSS 16.0 for Windows, IBM, Armonk, NY, USA), which was used to find the differential constituents for the classification between CMD and ZMD. According to the results of quantitative determination and *t*-test, the boxplots were charted by Origin pro 8 (Origin Lab, Northampton, MA, USA) to obtain an overview of the metabolite distribution and analyze the discrimination of CMD and ZMD. GRA can compensate for the shortcomings in statistical regression by Grey analysis, and be applied to evaluate the samples quality by measuring the approximate correlation between sequences. GRA was carried out to evaluate the quality of CMD and ZMD based on the contents of 32 constituents.

## 5. Conclusions

A reliable UFLC-QTRAP-MS/MS method was established to simultaneously determinate 32 components, including steroidal saponins, homoisoflavonoids, amino acids, and nucleosides, in Ophiopogonis Radix. Furthermore, multivariate statistical analysis including PCA, PLA-DA, *t*-test, and GRA were applied to comprehensively analyze and evaluate Ophiopogonis Radix from different habitats (CMD and ZMD). PCA, PLS-DA, and *t*-test were applied to classify and distinguish Ophiopogonis Radix from different producing areas. The results showed that CMD and ZMD were remarkably different, and the classification of them were related to the differential constituents, which could be considered as chemical markers to discriminate CMD and ZMD. Moreover, the GRA results demonstrated that the quality of CMD was better based on the contents of 32 constituents. These results suggested that the accumulation of bioactive constituents in Ophiopogonis Radix and the quality of this herb were influenced by the growth cycle and ecological environment. The research could not only provide a foundation for distinguishing CMD and ZMD, but lay the way for comprehensive evaluation and quality control of Ophiopogonis Radix from different areas.

## Figures and Tables

**Table 1:** Retention time, related mass spectrometry (MS) data of the target compounds.

No.	Compounds	t_R_ (min)	Precursor Ion (*m*/*z*)	Product Ion (*m*/*z*)	Declustering Potentional (DP)(V)	Collision Energy (CE)(eV)
1	Glutamic acid	0.52	147.08	83.92	83	14
2	Lysine	0.52	147.11	83.91	66	14
3	Histidine	0.53	156.08	110.03	95	16
4	Arginine	0.55	175.12	70.02	88	18
5	Threonine	0.57	120.07	74.00	93	20
6	Aspartic acid	0.57	134.05	87.96	59	10
7	Serine	0.57	106.05	59.99	67	8
8	Alanine	0.58	90.06	44.02	79	10
9	Proline	0.65	116.07	70.02	68	10
10	Valine	0.71	118.09	72.06	54	10
11	Cytidine	0.93	244.09	112.00	61	10
12	Uracil	0.98	113.04	70.00	111	21
13	Guanine	0.98	152.00	135.00	51	25
14	Methionine	1.02	150.06	104.03	91	10
15	2′-deoxycytidine	1.07	228.20	112.05	76	13
16	Isoleucine	1.36	132.10	86.05	64	10
17	Uridine	1.41	244.90	113.00	103	13
18	Leucine	1.48	132.11	86.05	98	10
19	Tyrosine	1.54	182.16	136.00	46	17
20	Adenosine	2.20	268.10	136.07	86	23
21	Inosine	2.47	269.00	137.07	46	15
22	Guanosine	2.52	284.30	152.00	62	15
23	Phenylalanine	3.04	166.10	120.05	56	14
24	2′-deoxyguanosine	3.16	268.10	152.10	61	15
25	Thymidine	4.57	243.10	127.07	61	13
26	Ophiopojaponin C	7.74	887.40	393.30	110	45
27	Ophiopogonin D	8.23	855.60	287.30	30	38
28	Ophiopogonin D’	8.30	855.60	253.30	130	45
29	Methylophiopogonone A	8.71	339.13	130.92	−135	−42
30	Methylophiopogonanone A	8.76	341.10	178.00	−110	−46
31	Methylophiopogonanone B	8.90	327.10	178.00	−90	−41
32	Ruscogenin	10.65	431.40	287.10	130	47

**Table 2:** Quality sequencing of Chuan maidong (CMD) and Zhe maidong (ZMD).

No.	*r*_i_	Ranking	No.	*r*_i_	Ranking
S1	0.5096	11	S15	0.6414	2
S2	0.5614	8	S16	0.6152	4
S3	0.6262	3	S17	0.3649	21
S4	0.4180	16	S18	0.3539	23
S5	0.4642	15	S19	0.3664	20
S6	0.4918	13	S20	0.3420	24
S7	0.5229	10	S21	0.3965	17
S8	0.4833	14	S22	0.3767	18
S9	0.5629	7	S23	0.3223	26
S10	0.6513	1	S24	0.2860	27
S11	0.5085	12	S25	0.3264	25
S12	0.5940	6	S26	0.3718	19
S13	0.6074	5	S27	0.3631	22
S14	0.5578	9			

**Table 3:** Information of Chuan maidong (CMD) and Zhe maidong (ZMD).

Samples	No.	Habitats	Samples	No.	Habitats
CMD	S1	Guanghui, Sichuan	ZMD	S17	Lishui, Zhejiang
	S2	Yongan, Sichuan		S18	Lishui, Zhejiang
	S3	Zengsheng, Sichuan		S19	Lishui, Zhejiang
	S4	Yongming, Sichuan		S20	Lishui, Zhejiang
	S5	Baiqing, Sichuan		S21	Lishui, Zhejiang
	S6	Lingxing, Sichuan		S22	Cixi, Zhejiang
	S7	Xinde, Sichuan		S23	Cixi, Zhejiang
	S8	Huanyuan, Sichuan		S24	Cixi, Zhejiang
	S9	Guangming, Sichuan		S25	Cixi, Zhejiang
	S10	Jianshe, Sichuan		S26	Cixi, Zhejiang
	S11	Songya, Sichuan		S27	Cixi, Zhejiang
	S12	Licheng, Sichuan			
	S13	Liuying, Sichuan			
	S14	Laoma, Sichuan			
	S15	Sanyuan, Sichuan			
	S16	Shimiao, Sichuan			

## Supplementary Materials

The following are available online. Figure S1: Chemical structures of 32 reference substances.

## References

[1] Chinese Pharmacopoeia Commission (2015). Pharmacopoeia of the People’s Repulic of China. Part I.

[2] Chen M.H., Chen X.J., Wang M., Lin L.G., Wang Y.T. (2016). Ophiopogon japonicus-A phytochemical, ethnomedicinal and pharmacological review. J. Ethnopharmacol..

[3] Wang L., Jiang X.L., Zhang W.M., Li F., Khan A.A., Liu X., Wang M.K. (2017). Homo-aro-cholestane, furostane and spirostane saponins from the tubers of Ophiopogon japonicas. Phytochemistry.

[4] Cheng Z.H., Wu T., Li L.Z., Liu N., Yu B.Y., Xu L.S. (2005). Studies on the liposoluble components from tuber of Ophiopogon japonicus. Chin. Pharmacol. J..

[5] Zhao J.W., Chen D.S., Deng C.S., Wang Q., Zhu W., Lin L. (2017). Evaluation of anti-inflammatory activity of compounds isolated from the rhizome of Ophiopogon japonicas. BMC Complement. Altern. Med..

[6] Ishibashi H., Mochidome T., Okai J., Ichiki H., Shimada H., Takahama K. (2001). Activation of potassium conductance by ophiopogonin-D in acutely dissociated rat paratracheal neurons. British. J. Pharm..

[7] He F., Xu B.L., Chen C., Jia H.J., Wu J.X., Wang X.C., Sheng J.L., Huang L., Cheng J. (2016). Methylophiopogonanone A suppresses ischemia/reperfusion-induced myocardial apoptosis in mice via activating PI3K/Akt/eNOS signaling pathway. Acta Pharmacol. Sin..

[8] Dong J.H., Pang X.C., Wang W., Wang Y.J., Li X.E., Qin M.J. (2015). Comparison on homoisoflavones extracted from Ophiopogon Japonicus in Sichuan and Zhejiang and their anti-oxidative activity. Chin. Tradit. Herbal. Drugs.

[9] Wang Y.C., Liu F., Liang Z.S., Peng L., Wang B.Q., Yu J., Su Y.Y., Ma C.D. (2017). Homoisoflavonoids and the antioxidant activity of Ophiopogon japonicus root. Iranian J. Pharm. Res. IJPR.

[10] Chen S., Li X., Liu L., Liu C., Han X. (2018). Ophiopogonin D alleviates high-fat diet-induced metabolic syndrome and changes the structure of gut microbiota in mice. FASEB J..

[11] Zhang H.Q., Liu P., Zhu Z.H., Dong L., Li W.W., Qian D.W., Duan J.A. (2017). Analysis and evaluation of amino acids and nucleosides in different parts of ripe fruit of Trichosanthes rosthornii harm. Mod. Chin. Med..

[12] Zhang H.Q., Liu P., Duan J.A., Dong L., Shang E.X., Qian D.W., Zhu Z.H., Li H.W., Li W.W. (2019). Comparative analysis of carbohydrates, nucleosides and amino acids in different parts of Trichosanthes kirilowii Maxim. by (Ultra) high-performance liquid chromatography coupled with tandem mass spectrometry and evaporative light scattering detector methods. Molecules.

[13] Lu X.Y., Tong W., Wang S.F., Li J.H., Zheng J., Fan X.H., Liu L. (2016). Comparison of the chemical consituents and immunomodulatory activity of ophiopogonis radix from two different producing areas. J. Pharm. Biomed. Anal..

[14] Li X.E., Wang Y.X., Sun P., Liao D.Q. (2016). Determination of saponin content in hang maidong and chuan maidong via HPLC-ELSD analysis. J. Anal. Methods. Chem..

[15] Wu Y., Dong Z.J., Wu H.Z., Ding W.J., Zhao M.M., Shi Q.W., Wang Q. (2014). Comparative studies on Ophiopogonis and Liriopes based on the determination of 11 bioactive components using LC-MS/MS and hierarchical clustering analysis. Food Res. Int..

[16] Jia C., Ye Z.L., Jiang X.J., Zhou D.Z., Li D.K. (2011). Simultaneous determination of contents of three flavonoid ingredients in Radix Ophiopogonis by HPLC. Chin. Pharmacol. J..

[17] Jiang Y., Duan C.Y., Chai X.Y., Hua H.M., Tu P.F. (2007). Studies on the chemical constituents of Ophiopogon japonicus. Chin. J. Chin. Mater. Med..

[18] Wu F.M., Cai X.Y., Wang P., Bao X.H., Li M., Zhou J. (2015). HPLC simultaneous determination of contents of 5 saponin constituents in Ophiopogonis Radix. Chin. J. Chin. Mater. Med..

[19] Wu F.M., Yang R.S., Zhang S.D., Bao X.H., Li M., Zhou J. (2016). Simultaneous determination of three flavone constituents in Ophiopogonis Radix by HPLC method. Chin. Pharm. J..

[20] Wang S.N., Hua Y.J., Xu L., Zou L.S., Liu X.H., Luo Y.Y., Liu J.X., Yan Y. (2016). Quality evaluation of Scrophulariae Radix processed by different ‘Sweating’ methods based on simultaneous determination of multiple bioactive constituents combined with grey relational analysis. Molecules.

[21] Chen C.H., Liu Z.X., Zou L.S., Liu X.H., Chai C., Zhao H., Yan Y., Wang C.C. (2018). Quality evaluation of Apocyni Veneti Folium from different habitats and commercial herbs based on simultaneous determination of multiple bioactive constituents combined with multivariate statistical analysis. Molecules.

[22] Yan Y., Zhao H., Chen C.H., Zou L.S., Liu X.H., Chai C., Wang C.C., Shi J.J., Chen S.Y. (2018). Comparison of multiple bioactive constituents in different parts of Eucommia ulmoides based on UFLC-QTRAP-MS/MS combined with PCA. Molecules.

[23] Chen S.Y., Shi J.J., Zou L.S., Liu X.H., Tang R.M., Ma J.M., Wang C.C., Tan M.X., Chen J.L. (2019). Quality evaluation of wild and cultivated *Schisandrae Chinensis* Fructus based on simultaneous determination of multiple bioactive constituents combined with multivariate statistical analysis. Molecules.

[24] Qu C., Pu Z.J., Zhou G.S., Wang J., Zhu Z.H., Yue S.J., Li J.P., Shang L.L., Tang Y.P., Shi X.Q. (2017). Comparative analysis of main bio-active components in the herb pair Danshen-Honghua and its single herbs by ultra-high performance liguid chromatography coupled to triple quadrupole tandem mass spectrometry. J. Sep. Sci..

