# Supplementary material for: Quality Evaluation of Ophiopogonis Radix from Two Different Producing Areas
Source: Molecules. 2019 Sep 4;24(18):3220. doi: 10.3390/molecules24183220 (PMC6766908; doi:10.3390/molecules24183220)
Supplement: Supplementary file 1 [file molecules-24-03220-s001.pdf]

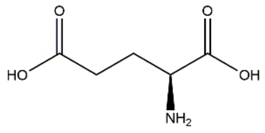
Glutamic acid (**1**)

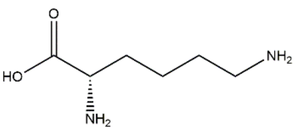
Lysine (**2**)

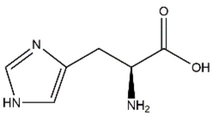
Histidine (**3**)

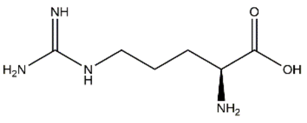
Arginine (**4**)

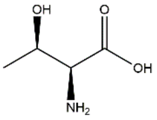
Threonine (**5**)

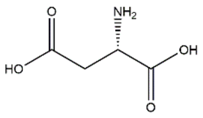
Aspartic acid (**6**)

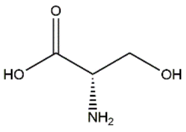
Serine (**7**)

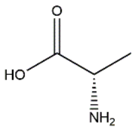
Alanine (**8**)

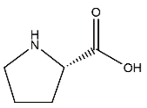
Proline (**9**)

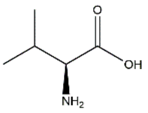
Valine (**10**)

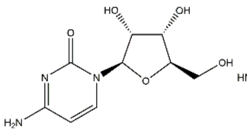
Cytidine (**11**)

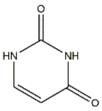
Uracil (**12**)

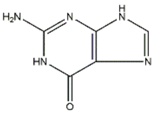
Guanine (**13**)

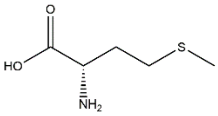
Methionine (**14**)

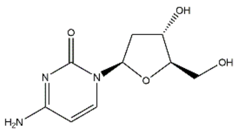
2′-deoxycytidine (**15**)

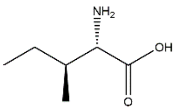
Isoleucine (**16**)

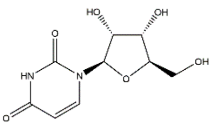
Uridine (**17**)

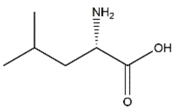
Leucine (**18**)

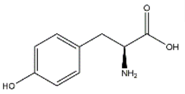
Tyrosine (**19**)

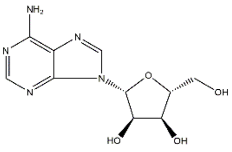
Adenosine (**20**)

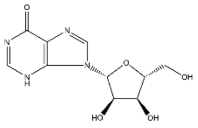
Inosine (**21**)

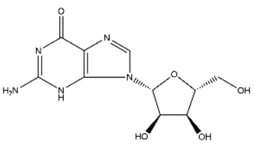
Guanosine (**22**)

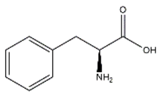
Phenylalanine (**23**)

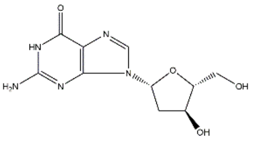
2′-deoxyguanosine (**24**)

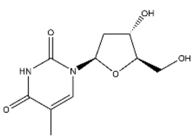
Thymidine (**25**)

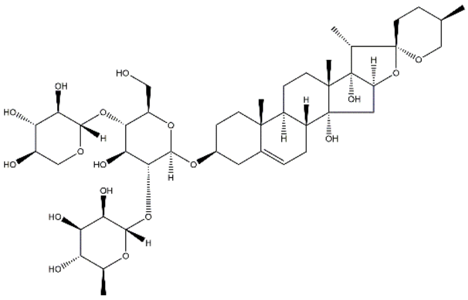
Ophiopojaponin C (**26**)

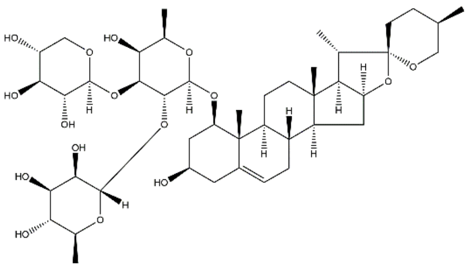
Ophiopogonin D (**27**)

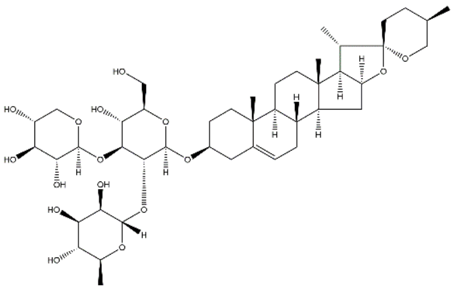
Ophiopogonin D′ (**28**)

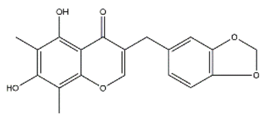

Methylophiopogonone A (**29**)

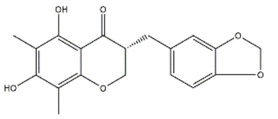

Methylophiopogonanone A (**30**)

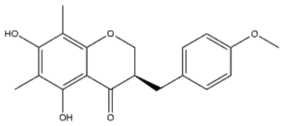

Methylophiopogonanone B (**31**)

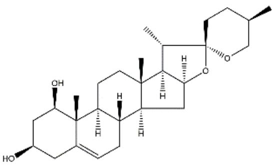

Ruscogenin (**32**)

Figure S1. Chemical structures of 32 reference substances

Table S1. Regression equation, limit of detection (LODs) and limit of quantification (LOQs), precision, repeatability, stability, and recovery of 32 investigated compounds.

No.	Compounds	Regression Equation	*r*	Liner Range (ng/mL)	LOD (ng/mL)	LOQ (ng/mL)	Precision		Repeatability	Stability	Recovery (%)		Matrix effect
							Intar-Day RSD (%) (n = 6)	Inter-Day RSD (%) (n = 9)	RSD (%) (n=6)	RSD (%) (n=6)	Mean	RSD	
1	Glutamic acid	$Y = 20.6X + 12300$	0.9992	7.60 - 76000	2.16	7.20	4.19	2.62	1.52	1.49	98.87	3.21	0.98
2	Lysine	$Y = 220X + 22000$	0.9999	3.05 - 30500	0.82	2.73	1.92	4.20	1.35	2.09	96.57	2.42	1.01
3	Histidine	$Y = 153X + 2410$	0.9996	0.47 - 4700	0.14	0.46	4.64	1.58	1.33	1.22	100.58	4.45	1.02
4	Arginine	$Y = 170X + 90400$	0.9993	74.9 - 749000	1.50	5.00	3.56	2.42	2.45	2.19	96.82	3.65	0.98
5	Threonine	$Y = 7.34X + 4190$	0.9995	14.60 - 146000	1.46	4.87	2.30	3.43	2.21	3.33	95.58	3.51	0.96
6	Aspartic acid	$Y = 76.8X + 3960$	0.9991	3.26 - 32600	0.33	1.10	3.71	4.43	3.17	3.59	97.76	3.59	1.02
7	Serine	$Y = 31.8X + 20600$	0.9996	7.54 - 75400	1.51	5.03	3.66	2.16	1.75	1.62	96.94	2.30	0.99
8	Alanine	$Y = 5.03X + 3390$	0.9996	73.10 - 731000	2.92	9.73	1.45	2.61	1.84	3.12	99.67	3.81	1.04
9	Proline	$Y = 801X + 24800$	0.9998	58.70 - 587000	1.17	3.90	3.44	4.80	3.41	4.31	97.85	4.13	0.98
10	Valine	$Y = 2440X + 112000$	0.9997	9.58 - 95800	0.96	3.20	2.07	2.87	2.91	2.36	96.77	3.40	0.97
11	Cytidine	$Y = 802X + 470$	0.9998	1.31 - 13100	0.30	1.00	2.67	4.69	2.84	2.98	97.21	3.52	1.05
12	Uracil	$Y = 16.8X + 1070$	0.9997	6.21 - 62100	1.43	4.77	3.62	2.66	2.96	2.85	98.46	3.34	1.01
13	Guanine	$Y = 311X + 5010$	0.9999	0.20 - 1970	0.19	0.63	2.53	4.57	3.71	4.05	95.63	3.18	0.98
14	Methionine	$Y = 27.8X + 301$	0.9995	1.05 - 10500	0.26	0.87	4.15	4.65	4.10	3.95	97.86	3.46	1.02
15	2′-deoxycytidine	$Y = 495X - 3180$	0.9994	7.03 - 70300	0.22	0.73	2.56	2.04	1.26	2.55	97.08	2.66	1.04
16	Isoleucine	$Y = 930X + 25300$	0.9994	0.78 - 7800	0.78	2.60	2.90	4.22	4.56	2.48	97.68	2.96	1.06
17	Uridine	$Y = 5.59X + 598$	0.9998	10.04 - 100400	1.04	3.47	3.59	3.09	1.18	1.64	98.11	3.25	0.99
18	Leucine	$Y = 27.7X + 8910$	0.9997	4.57 - 45700	1.32	4.40	3.44	3.32	3.74	3.53	96.88	3.98	0.96
19	Tyrosine	$Y = 580X + 18800$	0.9998	9.01 - 90100	0.90	3.00	2.64	2.53	2.17	1.88	98.17	2.44	0.98
20	Adenosine	$Y = 6300X + 129000$	0.9996	4.54 - 45400	0.44	1.47	2.66	3.89	3.77	3.42	101.04	3.32	0.93
21	Inosine	$Y = 1880X + 1400$	1.0000	1.75 - 17500	0.17	0.57	2.13	2.63	2.39	2.09	98.28	2.58	1.01
22	Guanosine	$Y = 349X + 3180$	0.9995	45.42 - 454200	0.45	1.50	3.74	2.33	1.49	1.76	101.92	2.25	1.03
23	Phenylalanine	$Y = 4320X - 47700$	0.9997	14.85 - 148500	0.74	2.47	2.18	4.22	2.66	3.99	100.53	3.01	0.96
24	2′-deoxyguanosine	$Y = 1010X + 456$	0.9997	1.50 - 15000	0.15	0.50	1.98	4.32	3.85	4.29	96.69	2.41	1.08
25	Thymidine	$Y = 35.3X + 477$	0.9996	0.44 - 4400	0.13	0.43	3.35	3.39	2.44	2.90	100.22	2.77	1.05
26	Ophiopojaponin C	$Y = 17.9X + 107$	0.9992	1.76 - 17600	0.45	1.50	2.25	4.24	3.98	3.53	99.38	1.89	0.95
27	Ophiopogonin D	$Y = 175X + 268000$	0.9996	36.80 - 368000	0.74	2.47	0.85	3.38	1.58	2.26	976.95	2.35	0.99
28	Ophiopogonin D′	$Y = 271X + 2270$	0.9997	2.06 - 20600	0.42	1.40	3.04	3.65	2.40	2.63	102.46	2.05	1.01
29	Methylophiopogonone A	$Y = 578X + 2360$	0.9992	0.63 - 6300	0.13	0.43	1.91	2.51	1.20	3.96	98.83	3.66	0.98

30	Methylophiopogonanone A	$Y = 2550X + 121000$	0.9992	4.93 - 49300	0.23	0.77	2.02	4.02	3.24	4.57	100.34	4.42	1.01	
31	Methylophiopogonanone B	$Y = 4730X + 91300$	0.9993	3.15 - 31500	0.11	0.37	0.67	2.24	1.12	3.18	99.54	1.29	0.99	
32	Ruscogenin	$Y = 5080X + 28100$	0.9998	0.64 - 6400	0.16	0.53	0.45	3.97	2.05	3.21	98.97	2.80	0.97	

Table S2. Contents of 32 compounds in Ophiopogonis Radix (μg/g, *n* = 3).

Analyte	CMD																ZMD										
	**S1**	**S2**	**S3**	**S4**	**S5**	**S6**	**S7**	**S8**	**S9**	**S10**	**S11**	**S12**	**S13**	**S14**	**S15**	**S16**	**S17**	**S18**	**S19**	**S20**	**S21**	**S22**	**S23**	**S24**	**S25**	**S26**	**S27**
1	184.20	226.20	228.00	158.70	192.60	164.40	205.80	181.80	248.40	263.70	178.80	233.10	240.30	216.00	244.20	215.70	65.34	30.30	35.10	2.50	45.00	64.50	114.90	21.12	78.30	21.50	7.86
2	84.30	84.30	99.00	72.60	71.70	67.20	93.60	79.20	84.60	92.70	75.60	90.30	93.00	73.80	97.80	89.10	2.61	15.45	24.45	8.34	18.84	40.20	53.70	15.12	42.60	30.12	15.75
3	19.44	24.90	27.81	16.56	22.68	21.60	24.36	23.04	23.82	27.45	27.96	23.28	25.89	20.64	27.21	33.90	12.78	21.03	8.49	11.55	21.57	12.33	11.34	9.87	14.67	26.83	18.96
**4**	1749.00	1743.00	2184.00	1590.00	1245.00	1500.00	1500.00	1761.00	1902.00	1671.00	1422.00	1764.00	1746.00	1899.00	2208.00	1716.00	318.00	789.00	837.00	348.00	858.00	1149.00	870.00	351.00	744.00	714.18	462.00
**5**	876.00	804.00	924.00	1002.00	699.00	1014.00	1029.00	1035.00	780.00	777.00	732.00	711.00	753.00	750.00	753.00	1185.00	68.10	218.10	66.30	65.10	233.10	80.70	53.40	226.80	390.00	72.48	105.00
**6**	849.00	927.00	945.00	624.00	687.00	816.00	852.00	738.00	939.00	1038.00	846.00	960.00	999.00	876.00	1026.00	969.00	360.00	483.00	321.00	330.00	450.00	318.00	339.00	214.20	411.00	217.81	519.00
7	221.70	229.80	243.60	185.70	205.20	226.50	225.60	220.50	222.00	225.00	198.00	204.00	235.50	236.10	232.80	238.20	38.40	41.40	60.90	37.80	51.60	84.90	133.20	67.20	126.00	139.37	43.80
**8**	1158.00	1188.00	1509.00	879.00	1107.00	1164.00	1191.00	1080.00	1293.00	1467.00	1224.00	1284.00	1452.00	1191.00	1653.00	1452.00	242.10	381.00	429.00	193.80	441.00	435.00	471.00	339.00	474.00	263.18	265.50
**9**	306.00	390.00	375.00	196.80	235.50	293.10	384.00	262.20	294.30	420.00	324.00	357.00	402.00	321.00	372.00	393.00	627.00	627.00	339.00	594.00	708.00	381.00	412.50	466.20	462.90	648.89	858.00
10	105.30	118.80	144.30	74.40	96.30	115.80	116.40	95.70	121.80	156.30z	123.00	130.50	135.30	114.60	152.70	138.60	10.71	21.72	31.20	10.74	22.02	31.80	36.00	17.01	37.20	71.31	14.58
11	5.70	10.05	7.53	3.90	6.39	5.49	5.70	4.80	7.65	10.08	6.84	10.02	6.99	7.35	9.60	8.16	1.60	1.51	2.71	1.43	1.88	2.22	2.09	0.48	1.40	1.16	3.39
12	7.71	9.93	10.74	5.13	3.39	5.79	8.61	7.53	10.38	10.71	10.35	13.38	8.79	9.06	7.32	4.29	1.99	3.96	7.71	4.47	3.12	4.86	1.89	0.12	3.03	2.36	3.27
13	3.36	6.39	4.68	1.58	3.57	2.78	4.08	2.64	3.66	7.56	4.86	5.94	8.16	4.59	7.26	6.45	1.20	1.02	12.93	30.30	1.42	13.05	0.62	0.16	0.75	4.73	11.55
14	47.70	44.70	59.40	27.93	30.00	37.20	34.20	36.00	57.90	69.60	38.10	59.70	43.20	64.50	67.80	45.60	0.04	3.42	4.20	-	4.56	4.47	10.17	1.54	8.91	17.85	0.40
15	1.09	0.98	1.22	0.66	0.98	0.87	1.04	0.85	1.16	1.42	1.04	1.01	1.31	1.05	1.36	1.09	0.54	0.55	0.48	0.60	0.74	0.46	0.47	0.26	0.53	0.44	0.67
16	86.70	104.10	159.00	59.70	72.90	102.00	104.40	85.20	106.20	150.30	99.30	117.00	138.30	113.40	166.20	145.50	10.89	25.38	25.08	11.04	24.06	29.40	31.80	11.82	29.04	24.27	14.67
17	170.40	167.40	266.40	118.80	149.70	158.70	168.30	127.80	204.00	267.00	187.80	216.30	263.10	194.10	256.20	228.00	75.00	92.10	150.60	92.70	78.30	155.70	45.60	53.46	41.10	103.09	122.70
**18**	459.00	582.00	723.00	287.10	408.00	462.00	522.00	420.00	672.00	834.00	513.00	678.00	732.00	597.00	795.00	678.00	73.50	141.30	217.80	69.90	155.70	234.90	118.80	41.10	114.90	86.07	115.80
19	176.70	221.40	276.90	118.20	147.60	183.00	190.20	169.20	227.40	276.90	194.70	220.50	249.90	231.00	288.60	255.30	24.99	54.00	48.30	25.98	56.40	52.20	52.20	19.53	59.10	63.86	36.90
20	57.90	64.20	82.80	39.60	46.20	49.80	54.30	53.40	79.20	85.20	51.00	77.40	82.80	73.50	76.80	75.60	13.56	36.60	41.40	13.92	34.80	45.30	16.44	5.46	13.65	14.32	18.78
21	1.78	2.91	3.21	1.12	1.76	2.33	2.06	1.73	1.86	3.90	2.08	2.93	3.39	1.74	3.33	3.03	2.27	1.88	1.80	2.02	1.98	2.07	0.36	0.43	0.49	2.54	2.22
**22**	187.20	219.00	297.90	143.10	182.10	173.40	186.30	182.10	275.70	306.00	178.20	267.00	271.50	249.30	299.70	270.60	13.71	50.40	120.90	15.57	51.90	130.20	55.50	16.59	48.30	38.56	24.99
23	75.00	77.40	110.10	49.20	56.70	69.60	72.60	69.30	93.90	105.60	68.10	93.30	98.40	98.70	106.50	100.50	5.94	12.03	20.16	6.15	12.45	21.12	20.61	6.78	22.98	29.52	9.09
24	3.60	5.55	5.97	2.33	3.99	4.23	3.84	3.24	3.96	5.97	3.48	5.34	5.10	4.53	5.85	5.70	0.35	1.22	1.34	0.34	1.48	1.39	1.27	0.36	1.06	1.10	0.77
25	11.10	9.78	11.64	1.29	8.64	7.53	8.76	4.11	9.36	12.36	9.54	13.68	14.25	6.72	17.22	15.78	7.92	3.09	6.03	7.68	2.63	4.89	0.59	0.53	0.83	1.71	6.42
26	39.60	47.40	59.40	28.29	34.80	40.20	48.90	34.50	40.80	60.60	42.90	45.00	50.10	46.80	52.20	70.50	85.50	57.30	18.15	57.00	85.50	57.30	8.64	39.00	14.13	11.87	57.00
27	117.90	150.60	131.10	68.10	69.30	78.00	117.60	108.30	147.60	168.90	86.40	178.20	156.00	160.80	149.40	167.10	-	-	-	-	-	-	-	-	-	-	-
28	7.14	6.81	10.29	4.65	5.88	4.53	6.60	5.82	7.08	12.48	7.14	8.01	10.23	9.48	9.39	10.02	1.36	1.41	1.97	0.86	1.36	1.41	0.83	0.92	1.56	6.61	0.86

29	1.17	1.97	1.93	0.79	1.10	1.40	1.46	1.19	1.55	2.52	1.11	2.25	1.91	1.86	2.29	2.83	3.93	1.33	0.99	2.39	4.05	1.33	1.50	0.41	0.52	2.49	2.42
30	41.70	48.30	56.70	28.65	30.90	37.80	39.60	44.40	48.90	52.80	25.95	52.80	51.90	57.90	50.10	60.90	229.20	127.80	214.20	167.70	229.20	127.80	64.50	151.50	248.40	65.52	167.70
**31**	16.92	22.47	25.47	11.37	13.68	18.84	17.61	19.56	21.36	25.62	13.32	24.42	23.25	24.18	24.63	29.52	393.00	240.60	175.80	306.00	393.00	240.60	280.20	63.60	110.40	38.37	306.00
32	0.43	0.56	0.62	0.30	0.33	0.37	0.50	0.44	0.57	0.77	0.50	0.72	0.75	0.67	0.68	0.77	1.74	1.34	5.58	0.40	1.74	1.34	-	0.07	0.06	-	0.40

Note: “-”not detected.
